# Exploring the Role of Alignability Effects in Promoting Uptake of Energy-Efficient Technologies

**DOI:** 10.1037/xap0000253

**Published:** 2019-11-14

**Authors:** Rebecca J. Hafner, David Elmes, Daniel Read

**Affiliations:** 1 Department of Psychology, University of Bath; 2 Warwick Business School, University of Warwick

**Keywords:** alignability effects, decision-making, energy demand reduction, energy-efficient technologies, consumer behavior

## Abstract

The current research applies decision-making theory to the problem of increasing uptake of energy-efficient technologies, where uptake is currently slower than one might predict following rational choice models. We explore the role of alignability effects on consumers’ preference for standard versus energy-efficient technologies. Previous research has found that attentional weight given to alignable or nonalignable features varies depending on the decision context, including between-alternative heterogeneity. In a hypothetical choice task, subjects were presented with a choice between similar (boiler vs. boiler) versus dissimilar (boiler vs. heat pump) home heating technologies, each described by a list of alignable and nonalignable attributes. We found a preference for alignability when options were similar; an effect mediated by an increased tendency to infer missing information is the same. No effects of alignability on preference were found when options differed. We draw theoretical and applied implications for (a) the role of alignability effects in contributing to the energy efficiency gap and (b) the type of information structure best suited for the promotion of energy-efficient technologies in future marketing campaigns.

## Public Significance Statement

We explore the role of alignability effects in determining preference for green versus nongreen technologies, and find that attention appears to be divided between alignable and nonalignable features when options differ. We discuss implications for real-world application in terms of promoting uptake of energy-efficient technologies, and thus reducing the energy efficiency gap in this context.

Promoting consideration and uptake of technologies designed to reduce energy demand is a key societal challenge (Ölander & Thøgersen, 2014; Steg & Vlek, 2009). Research has established that a vast number of psychological barriers, such as normative influence, action inertia, and habit may prevent people from acting in proenvironmental ways (American Psychological Association, 2009; Dolan, Hallsworth, Halpern, King, & Vlaev, 2010; Nolan, Schultz, Cialdini, Goldstein, & Griskevicius, 2008; Pichert & Katsikopoulos, 2008; Schultz, Nolan, Cialdini, Goldstein, & Griskevicius, 2007). This includes the purchase of green, or energy-efficient, products versus standard counterparts (Hafner, Elmes, & Read, 2019; Jager, 2006; Steg & Vlek, 2009), where uptake is typically slower than one might predict following rational choice models (Lindenberg & Steg, 2007); an effect which has been termed the “energy-efficiency gap” (Jaffe & Stavins, 1994). It is suggested that changing purchase behavior may have greater environmental benefit than reusing or recycling available products (see Steg & Vlek, 2009). Consequently, overcoming these barriers to change consumer purchase behavior and reduce the energy efficiency gap is a key goal in the strive toward EU2030 carbon dioxide (CO_2_) emissions targets (Committee on Climate Change, 2011; Steg, Perlaviciute, & van der Werff, 2015; Stephenson et al., 2010).

A substantial body of research in psychology and behavioral science has identified that a key factor contributing to preference formation and product selection concerns the ways in which options or choice sets are initially presented or framed. Research into alignability effects has shown, for instance, that people may be more or less attracted to options according to variations in structure of information and framing of descriptive features provided (Xie, Mattila, & Kerstetter, 2011; Zhang & Markman, 2001). The main aim of the current research is to extend this research into the novel domain of new technology adoption, with a view to determining how the choice environment can most effectively be structured to promote consideration of such technologies, and thus reduce the energy efficiency gap. We explore this with a hypothetical choice experiment using the example of heating system choice. However, the principles covered apply to all such technologies, and so may be of interest to researchers looking to encourage green decision-making across any number of contexts. We begin by providing a discussion of research into alignability effects, before moving on to discuss the how this may contribute directly to the continued prevalence of the energy-efficiency gap, and thus how developed understanding of this influence on consumer decision-making may help promote choice of green versus nongreen technologies.

## Option Alignability

Much research in psychology and behavioral science has explored the impact of informational structure and attribute relationships in the formation of preferences. This line of research stems largely from Tversky’s (1977) model of similarity and contrast, which asserts that people represent information about available options and their attributes as a set of features. These features can then be compared, allowing decision makers to determine commonalities and differences upon which to base subsequent preference judgments.

How people evaluate options depends not just on what information is provided, but also on how the different features are presented. For instance, features can be made either directly comparable with other options or not. Features which are shared by all alternatives are referred to as commonalties, whereas unique features can be broadly defined as being either alignable or nonalignable (Gentner & Markman, 1994, 1997). Alignable features are defined as having varying levels of the same feature, while nonalignable attributes are not shared by all alternatives (Gentner & Markman, 1994, 1997). For example; providing information on the (different) calorie contents of two snacks would make calorie content an alignable feature, yet if the information on calorie content was identical for both options this would make calorie content a commonality. On the other hand, describing one option as having low levels of salt, with no corresponding information available for the second option, would make salt level a nonalignable feature, for which no direct comparison is available.

Research has explored the comparative impact of these shared versus unique features in decision-making; typically finding that features which are common to all options available within a choice set often have little to no bearing on subsequent preference judgments. In effect, these similar features, or *commonalities*, are typically seen to cancel each other out (Houston & Sherman, 1995), given that choosers inevitably end up acquiring the shared features, regardless of whichever option they select. Accordingly, research has shown that decision makers’ typically place more weight on *unique* features, downplaying or overlooking shared or common features. For example, in Houston and Sherman’s (1995) study, participants had to choose between pairs of goods (such as automobiles), which either shared good features (e.g., stereo included) and had unique bad features (such as poor mileage), which were termed *unique-bad* pairs, or shared bad features, and had unique good features (*unique-good* pairs). The authors found that in each instance, the common features were effectively cancelled out, leading to greater postchoice evaluations of alternatives judged from unique-good pairs, that is, those with distinctive positive features (see also, Slovic & MacPhillamy, 1974). This finding has even been applied to intertemporal choice, showing there is little discounting for options that differ by many features including delay, but a great deal of discounting for options that differ by few features apart from delay (Cubitt, McDonald, & Read, 2018).

The difference between alignable and nonalignable features is central to the structural alignment model of similarity (Gentner & Markman, 1997). According to this model, when valence of features is not directly manipulated, alignable features will generally be given greater weight during comparison tasks. Many studies have found results consistent with this. For example, Slovic and MacPhillamy (1974) found that when making predictions people focus more on information provided for all options than on information available for one option only. Similarly, Markman and Medin (1995) found that people were more likely to justify their decisions by referring to alignable versus nonalignable features. In their experiment, participants were asked to rate their preference for pairs of video games which varied in their direct comparability. When asked to explain their decisions, justifications were found to systematically favor comparable over noncomparable properties, providing evidence of a constructive alignment process central to judgments of similarity in choice (Markman & Medin, 1995).

However, research has also found that the attentional weight given to alignable versus nonalignable features can vary according to shifts in the decision context. For example, Zhang and Markman (2001) showed that how involved people were in the task moderated the effects of attribute alignability. They presented participants with a choice of popcorn brands, defined according to various alignable and nonalignable attributes, and asked them to select the most attractive option. Level of task involvement was manipulated by either informing participants that their data would be combined with that from thousands of other respondents, to get a sense of the average consumer (low-involvement), or that they were one of a select group chosen to participate, and that their responses would be crucial in the development of products due to be launched in the area very soon (high-involvement). Respondents were found to focus more on alignable attributes in low- versus high-involvement conditions. Zhang and Markman (2001) suggest that this effect occurs because nonalignable features require greater effort to process, and that respondents only deploy that effort when they are highly involved in the task.

Option *similarity* has also been shown to impact on preference for alignable versus nonalignable features. In an experiment conducted by Xie et al. (2011), participants were presented with a choice of two popcorns, which were either low in between-alternatives heterogeneity—as they were both made by the same company, or high in between-alternatives heterogeneity—as they were made by different companies. Xie et al. found that consumers attached more importance to alignable attributes when between-alternatives heterogeneity was high, and more on nonalignable attributes when this heterogeneity was low. They argued that when heterogeneity is high, consumers face high uncertainty which alignable attributes can reduce. On the other hand, when heterogeneity is low, the consumer’s main goal is discrimination between options, and nonalignable attributes serve this goal because they are more distinctive and discriminative. Overall, the research shows whether consumers focus on alignable or nonalignable features depends on variations in the construction of the choice environment.

## Alignability Effects and the Energy Efficiency Gap

The current research extends this line of research into the specific domain of energy choice, using the example of home heating system choice. This is a useful extension of previous research into alignability effects, which has typically focused on smaller scale consumables, given that alignability effects may contribute to the continued prevalence of the energy efficiency gap with large-scale product purchase decisions. Specifically, we theorized that given how previous research has demonstrated that people typically attach greater value to alignable features when options differ (Xie et al., 2011), it is possible that when considering new technologies which differ from the standard or norm, this increased tendency to focus on alignable features (such as upfront and operating costs) may explain continued preference for standard nongreen products. By extending this line of research into the context of large-scale green purchase decisions, we explore whether varying weight attributed to alignable versus nonalignable features contributes to the energy-efficiency gap, and thus determine whether variations in attribute framing can be used to promote new technologies.

In the U.K. domestic market, consumers typically use a gas-burning furnace, or *boiler*, for heating. A more energy-efficient technology would be a heat pump. In our hypothetical choice experiment, we presented participants with a choice between same (boiler vs. boiler) versus different (boiler vs. heat pump) technologies, each described by a list of attributes that are randomly assigned to be either alignable or nonalignable. In the interests of consistency with previous work, a snack choice task was also incorporated into the design, in which participants were given a choice between same (popcorn vs. popcorn) versus different (popcorn vs. pretzels) products. We aimed to determine whether previous results generalize to decision contexts with potentially wider-reaching implications (i.e., selection of new vs. standard technologies), or are restricted to the more commonly used context of snack choice.

Following the procedures used in previous research (Malkoc, Zauberman, & Ulu, 2005; Xie et al., 2011; Zhang & Markman, 1998, 2001), in each instance one option was presented as having superior alignable features, while the other was presented with superior nonalignable features. As far as we are aware, no previous studies have randomized which option was superior on alignable versus nonalignable features. In addition, ours is the first study that we know of which randomly allocated attributes to be alignable or nonalignable. More detail on this is provided in the Method section.

## Information Inference Processing

Another strand of choice research which has been subject to considerable interest in recent years concerns how consumers infer missing information. It is widely accepted that people form inferences about missing information on the basis of the information they have available, and that this information is used in subsequent preference judgments (see Dick, Chakravarti, & Biehal, 1990; Simmons & Lynch, 1991). This strand of research is particularly relevant to studies of alignability effects in which decision makers are presented with partial information about one or more alternatives in nonalignable choice subsets. Perhaps it is somewhat surprising then that the explicit link between the two literatures remains unexplored. It may be that differences in inference processing strategies can help to explain shifting focus on alignable versus nonalignable attributes in alternate decision circumstances. The current research aims to address this possibility.

Research by Ross and Creyer (1992) demonstrated that inferred values of missing nonaligned information are directly related to variation in other brand information. In this study, high variation in other-brand information on aligned attributes was found to result in a significantly discounted inferred value for the nonaligned attribute in question, whereas when other-brand variation was low, the inferred value was not discounted for uncertainty. However, to date no studies, as far as we are aware, have explored whether a difference in inference-processing strategies can help to explain the shifting preference for options with alignable versus nonalignable features (e.g., Xie et al., 2011, Zhang & Markman, 2001). We address this lacuna by comparing inference processing using similar versus dissimilar products in which the alignability of features is directly manipulated, allowing us to explore the potential for alternate inference-formation processing strategies to explain the varying attentional weight given to alignable or nonalignable features when products are similar vs. dissimilar.

## Hypotheses

Based on previous research into the effects of option similarity on alignability preference (Xie et al., 2011), we predicted that when presented with similar options (i.e., two boilers), the probability of selecting whichever option has superior nonalignable features would be increased. Yet, when presented with different options (boiler vs. heat pump) this preference would be reversed, and there would instead be an increased probability of selecting whichever option had the superior alignable features. Thus, in terms of the main focus of the current research, it was predicted that by manipulating attribute alignability, and presenting the green (vs. ‘standard’) option as having comparably superior alignable features, we may be able to encourage selection and uptake of innovative green technologies. In addition, on the basis of previous research into inference process modeling (Ross & Creyer, 1992), we also aimed to test an explanation for this effect—that more weight may be given to alignable features when options are similar versus dissimilar because of an increased tendency to infer that missing nonaligned information is the same when options are similar (i.e., there will be an increased tendency to copy any missing information over for nonalignable features when options are similar vs. dissimilar).

## Method

### Participants

Participants (*N* = 206; 78 male, 128 female, age range 18–54) recruited via the University of Warwick’s internal participant pool took part in the study in exchange for enrolment in a prize draw to win one of twenty £20 Amazon vouchers.

### Design

The study had a 2 (Similarity: similar vs. dissimilar) × 2 (Task: snacks vs. heating system), design with repeated measures on the last factor, and with likelihood of selecting the superior alignable versus nonalignable option as the main dependent variable. Ninety-five participants were given a choice between similar products (boiler vs. boiler); 111 were given a choice between dissimilar products (boiler vs. heat pump). Ethical approval for the experiment was granted by the humanities and social sciences internal research ethics committee at The University of Warwick.

### Manipulating Attribute Alignability

Following the procedure devised by Zhang and Markman (1998, 2001), paired products were described by a list of 12 features: four alignable features, four nonalignable features, and four commonalities. Previous work has largely allocated the same features to be either alignable or nonalignable across conditions, meaning it is virtually impossible to determine whether the effects found are attributable to experimental manipulations or to the relative importance of each feature type. For example, Zhang and Markman (1998, 2001) always use calorie content as an alignable feature, which may in fact hold greater sway during decision-making than some of the assigned nonalignable features.

To overcome this, we devised a means of randomly allocating each attribute to be used interchangeably as either alignable or nonalignable. A 3-point scale of response was devised for each attribute, where each had a clear best possible and worst possible response. This meant attributes could be used interchangeably as either superior or inferior features. Descriptions were provided for all attributes that were not self-explanatory. For example, the attribute *efficiency rating*, was described as follows: “Measured by an independent laboratory, this is the measured energy efficiency of the appliance, including heat and hot water; it is calculated to an industry standard called SAP2009. This SAP rating is expressed on a scale of 1 to 100; the higher the number, the lower the running costs.” To increase the ecological validity of the task, the attributes used in the heating system choice were adapted from review websites such as Which? (2018). Full details of all of the attributes used are presented in Appendix A.

**Figure 1 fig1:**
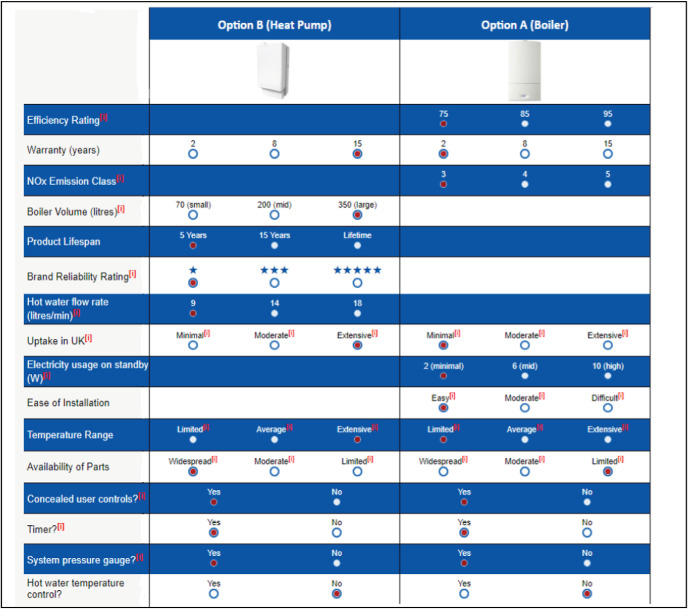
Sample screenshot of the heating systems choice platform.

In addition, participants were shown the full three-option scale of response for each attribute at the moment of choice, making it possible to determine how each feature rating related to the best or worst possible outcome on each given scale. So in the case of the attribute *efficiency rating*, subjects were told there were three possible levels of response, with 75 being the lowest (i.e., worst) possible outcome and 95 being the highest (i.e., best) possible outcome. In this manner, subjects were then able to easily compare the values shown with best and worst possible outcomes on each scale. These scales of response were each determined from assessment of products currently available on the marketplace. Figure 1 provides a sample screenshot of the choice platform.[Fig-anchor fig1]

As can be seen from Figure 1, if used as a nonalignable feature, information was provided for one option only. However, if the feature was alignable then values were provided for both options. In each case, one option was always presented as the superior alignable option, and was given the best possible scaled response for each of these attributes, whereas the other was presented as the superior nonalignable option, and given the worst response on the same (alignable) scale. Simultaneously, for the nonaligned features, the superior alignable option was given the worst possible response on provided scales, whereas the superior nonalignable option was given the best possible response for each nonalignable feature.

The attributes used in the snack choice task were kept as near as possible to those used in previous work (Xie et al., 2011; Zhang & Markman, 1998, 2001). However, in some instances, these were altered so that each could be presented with a 3-point scale of response (e.g., original feature of *pops in its own bag* vs. *requires a microwave bowl* could not be given a sliding scale of response and so was substituted), and so that the features could be used interchangeably to describe both popcorn and pretzels (full details of all the attributes used in this task are provided in Appendix B). Following the above procedure, it was then possible to randomly allocate attributes as either alignable or nonalignable and as superior or inferior, allowing for a fully controlled exploration of the effects of product similarity on alignability preference, which overcomes potential design effects of previous studies.

### Procedure

The order of tasks (heating system vs. snack choice) was counterbalanced. Participants were randomly allocated to one of two (similar vs. dissimilar products) conditions for each task, and were instructed:

We are exploring how people compare products on the web. Please imagine you are about to buy a new heating system for your home/Please imagine you are about to choose a snack to eat. You have narrowed your choice down to the two options, which will be presented side by side on the next screen. Please take your time to carefully consider these provided options. Once you have read the provided information, you will be asked to select which of the two options you will select for your heating system/snack. Each option is described according to information provided by the manufacturer.

Similar condition(s): Both are standard condensing boilers, which are fuelled by gas/both are popcorns.

Dissimilar condition(s): One is a standard condensing boiler, which is fuelled by gas. The other is a heat pump, which captures ambient heat from the ground and transfers it inside a building using mechanical energy/one is a type of popcorn, the other a type of pretzels.

Participants rated which option they preferred on 4-point scale ranging from 1 (*strongly prefer option A*) to 4 (*strongly prefer option B*). This measure was designed to provided an indication of preference formation based upon the information provided within the choice scenario. Previous research in applied decision-making has consistently shown that hypothetical scenarios such as this can provide a valid indication of the impact of experimental manipulations on both preference formation and behavior intentions (i.e., intention to carry out the behavior, or purchase a particular product, in real life). This has been widely illustrated using a variety of choice contexts, including both heating system choice and food choice (see, e.g., Chernev, 2008; Hafner, Elmes, Read, & White, 2019; Xie et al., 2011; Zhang & Markman, 1998, 2001). Consequently, although more research will undoubtedly be needed to generalize our findings, we nevertheless expected to see a difference between conditions according to the experimental manipulations used in our hypothetical choice tasks.

Finally, to test our hypothesis that greater weight may be placed on alignable features when options are similar versus dissimilar because of an increased likelihood of inferring nonaligned information is the same, we measured participants’ inferred responses for each nonaligned feature. This was done using the following instruction:

Some information was missing from the descriptions of the two options. You will be asked to think about this missing information and to ‘fill in the blanks’ of missing information using the options provided below. Please simply add the information you feel is most likely to fit into the missing sections.

Following the procedures used in previous studies of inference processing, participants were required to fill in the blanks for each nonalignable feature. For example, Ross and Creyer (1992) presented subjects with a choice among different alternatives, each described by a list of features, for which some information was missing. Subjects were asked to provide an inference for the missing value, as well as rating their preference among the alternatives. We replicated this element of Ross and Creyer’s (1992) methodological approach, with likelihood of copying versus not copying information from the corresponding nonaligned variable forming our primary mediator variable. This enabled us to assess the likelihood that respondents would be more likely to fill in the blanks by directly copying missing nonaligned information when options were similar versus dissimilar. The preference selection and fill in blanks tasks were counterbalanced, with half the respondents first indicating which option they preferred, and half first stating the values of missing variables.

## Results

### Option Preference

All respondents were given one superior alignable option and one superior nonalignable option. The main dependent variable (DV) was choice of the option superior versus inferior on the alignable/nonalignable features dimension. This DV was termed *alignability preference* and was coded as −1 (prefer option with superior nonalignable features) versus 1 (prefer option with superior alignable features). To test our hypothesis that when options were similar, alignability preference would be lower (i.e., there would be a preference for options with superior nonalignable features), an independent samples *t* test contrasting similar (1) and dissimilar (−1) groups was conducted. For the heating system choice task, *t* tests revealed a significant main effect of similarity on alignability preference: *t*(204) = 5.87, *p* < .001, with Cohen’s value (*d* = .83) representing a large effect size.

Breaking this down further to test our specific hypotheses for the differing impacts of alignability focus when products were similar versus dissimilar, one-sample *t* tests revealed that when options were similar, respondents were primarily driven by aligned features, with 86.3% of respondents selecting whichever option had superior alignable features. Conversely, when faced with dissimilar products, 49.5% preferred the superior alignable option. The effect of similarity on alignability preference was found to be significant for similar, *t*(94) = 10.25, *p* < .001, *d* = 1.05 (representing a large effect size), but not dissimilar, *t*(110) = .09, *p* = .93 products, *d* = .009.

Crucially, in terms of our primary focus on choice of green versus nongreen technologies, it did not matter which of the two options was presented as having superior alignable features when the options differed; when the boiler was presented as having superior alignable features, 49.1% of subjects chose this option. Conversely, when the heat pump was presented as having superior alignable features, 55.4% chose this option. What is more, *t* tests revealed there was no difference in preference for green versus nongreen products according to feature alignability: *t*(109) = −.47, *p* = .64, *d* = .09. Thus, in contrast to our predictions, when options differed, feature alignability appeared to be of reduced significance in terms of preference formation. We later return to a discussion of the implications of this finding.

**Figure 2 fig2:**
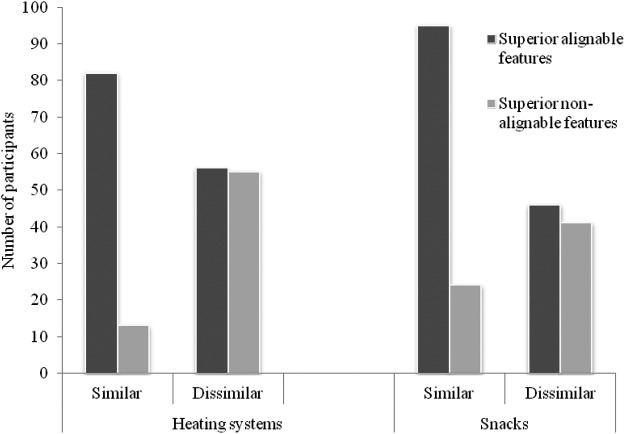
Bar chart displaying the effect of similarity on alignability preference for similar but not dissimilar products, for both the heating system and snack choice tasks.

Results were paralleled in the snack choice task, providing useful verification of the methodological procedure. Specifically, once again a main effect of similarity on alignability preference was found: *t*(204) = 4.27, *p* < .001, with Cohen’s value (*d* = .59) representing a medium effect size. Respondents were significantly more likely to select whichever option had the superior alignable features when products were similar: (79.8%) *t*(118) = 8.08, *p* < .001, *d* = .74, representing a large effect size. Yet no effects of alignability preference were found when options were dissimilar: *t*(86) = .53, *p* = .60, *d* = .06, with 52.9% selecting the superior alignable option. Results for both tasks are presented in Figure 2.[Fig-anchor fig2]

### Mediation Analyses

On the basis of previous research into inference-formation processing (Ross & Creyer, 1992), we predicted that any preference for options with superior alignable features might be explained by an increased tendency to infer that any missing feature information on one option is the same as that on the other option. To investigate this prediction we first calculated the average likelihood to replicate information for nonalignable features both for the snack choice and heating system choice tasks. For each of the eight nonaligned features provided (i.e., four for each product), any response directly copied from one option to the other was coded as 1 and any different response (either of the other two possible options on the response scale) coded as 0. Results are displayed in Table 1.[Table-anchor tbl1]

**Figure 3 fig3:**
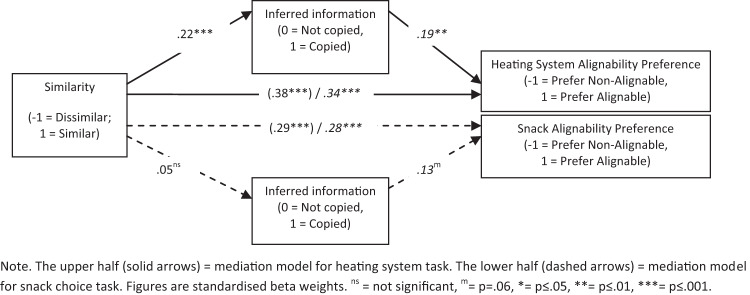
Mediation analysis showing the role of inferred information in mediating the effect of similarity on alignability preference for heating system choice, but not snack choice.

We then conducted two three-step mediation models to explore the role of inferred information as a potential mediator of the effects of similarity on alignability preference. Following Baron and Kenny (1986) we regressed: (a) similarity (similar vs. dissimilar) on alignability preference, (b) similarity on inferred information (copied vs. not copied responses), and (c) both similarity and inferred information onto alignability preference. The results are summarized in Figure 3 with the results from Step 1 shown in brackets and those from Step 3 in italics. The upper half of Figure 3 shows the mediation model for heating system choice, while the lower half shows the model for snack choice.[Fig-anchor fig3]

Heating system choice—Step 1 replicated earlier alignability analyses with increased preference for superior alignable option(s) in the similar versus dissimilar product condition (*M*s = .73 vs. .009; β = .38, *p* < .001). Step 2 found respondents were more likely to copy when presented with similar versus dissimilar products (*M*s = .37 vs. .27; β = .22, *p* = .001). Step 3 suggests that likelihood of inferring information is the same from one option to the next affected alignability preference irrespective of condition (β = 2.93, *p* = .004). The main effect of similarity on alignability preference remained significant (β = 5.19) once inferred information data were added to the model, nevertheless a Sobel test confirmed that inferred information was a significant mediator of the effects of similarity on alignability preference (*z* = 2.14, *p* = .003, two-tailed).

The same approach to analyzing the role of inferred information as a potential mediator of the effects of similarity on alignability preference was then carried out for the snack choice task. As before, Step 1 replicated earlier alignability analyses with increased preference for superior alignable option(s) in the similar versus dissimilar product condition (*M*s = .60 vs. .06; β = .29, *p* < .001). Step 2 found that there was no difference in inferred information when presented with similar versus dissimilar products (*M*s = .30 vs. .28; β = .05, *p* = .44). Step 3 suggests a marginal effect of inferring same information affected alignability preference irrespective of condition (β = .13, *p* = .06). The main effect of similarity on alignability preference remained significant (β = .28) once inferred information data were added to the model. A Sobel test confirmed that inferred information did not mediate of the effects of similarity on alignability preference for this task (*z* = .77, *p* = .44, two-tailed).

## Discussion

We explored the impact of attribute alignability effects on option preference, with a view to determining how the choice environmental might be structured to promote consideration and uptake of new green technologies. In line with the structural alignment model of similarity (Gentner & Markman, 1997), we found evidence that people focused more on alignable features when the options were similar (i.e., two boilers or two types of popcorn). However, in contrast to some previous research (Xie et al., 2011), our findings suggest that alignable and nonalignable features were given equal weight when the options are dissimilar (i.e., a boiler vs. heat pump, or popcorn vs. pretzels).

By combining the alignability and inference formation literatures we also found evidence for a novel explanation for this effect—that people are more likely to perceive that nonalignable information is the same when options are similar versus dissimilar. This was revealed by the fill in the blanks element of the choice task, which demonstrated that there was an increased tendency to copy missing nonaligned information over when contrasting similar (boiler vs. boiler) versus dissimilar (boiler vs. heat pump) products, an effect which was found to mediate the effects similarity on alignability preference for the heating system task. Thus, our results provide initial evidence to suggest that decision makers may not use the same processing strategy when assessing similar, familiar technologies versus more unfamiliar, dissimilar alternatives. Specifically, when choosing between similar options, it appears that nonaligned features may effectively be perceived as commonalities, thereby explaining reduced focus on these features which are known to be typically cancelled out during decision-making (Houston & Sherman, 1995). On the other hand, when considering dissimilar options, people are less likely to perceive missing information as the same from one option to the next, and thus attention is divided between the alignable and nonalignable features as both are perceived as providing a unique contribution to the weighing up process (i.e., neither type is perceived to effectively constitute a commonality).

Interestingly, some research has suggested that inferences about missing information do not occur unless they are prompted (see, Huber & McCann, 1982). As such, directly prompting subjects to infer missing information could have biased the choice results on this inference task, and we recognize this as a potential limitation of the current research. Yet, by counterbalancing the preference selection and fill in the blanks tasks we negated the potential impact of any such order effects, given that half of the participants made their selection before, and half after, having inferred missing information for nonaligned features. As such the current research provides evidence to counter these earlier claims (Huber & McCann, 1982), by demonstrating that the effects of inference processing on preference formation appear to occur regardless of whether subjects have been previously explicitly prompted to make such inferences, or not.

What is more, our findings demonstrate that previous work which has found that people focus more on alignable features when options differ (e.g., Xie et al., 2011) may be at least partly attributable to design effects, given that ours is the first study to randomly allocate features to being alignable versus nonalignable. Previous work has relied upon predefined subsets of alignable versus nonalignable attributes, consequently making it difficult to determine whether the effects found are attributable to experimental manipulations or simply to the relative importance of attributes that are more readily defined as alignable or nonalignable. Indeed, when we do incorporate random allocation of attribute alignability, some of the findings of previous work (e.g., Xie et al., 2011) appear to be reversed, with a greater preference for superior-alignable options when options are *similar*. When options differ, we find no evidence of any impact on alignability preference: people were just as likely to pick the superior alignable versus nonalignable option, again providing an interesting point of contention with previous work. Either the findings of previous work (e.g., using same vs. different brands) do not generalize to the context of same versus different technologies, or perhaps again these previous findings may have more to do with design effects than a controlled exploration of the effects of similarity on alignability preference.

Our results have potentially important implications for reducing the energy efficiency gap for large-scale purchase of green purchase decisions. Specifically, when faced with a choice of novel technologies that may be typically regarded as being outside of the norm and which differ from standard options, our results suggest that consumers are more likely to attempt to attend to all of the information presented at the moment of choice. As previously discussed, this appears to represent a different processing strategy to that used when faced with a choice of similar goods, in which subjects primarily attend to *alignable* features, given the assumption that that nonalignable information is likely to be the same. So, why could this be the case? And what are the implications for reducing the energy efficiency gap? One potential explanation relates to the bounds on cognitive capacities for information processing first outlined by Simon (1947, 1956). Specifically, it may be that people are driven toward selecting options they are more familiar with when faced with a choice between standard versus new, more energy-efficient technologies as this represents a means of simplifying a potentially overwhelming choice set. In other words, assimilating information on both alignable and nonalignable features accompanying new technologies may simply be too cognitively demanding, thus leading to an increased likelihood of reliance on simplification strategies such as deferring to one’s own previous experience, or copying the behavior of others (American Psychological Association, 2009; Dolan et al., 2010; Gilovich, Griffin, & Kahneman, 2002; Hafner, Elmes, & Read, 2019; Schwartz, 2000, 2004; Thaler & Sunstein, 2008); all of which are likely to avoidance of selecting goods which are outside of the norm.

Consequently, our results suggest that alignability effects impact product choices, as demonstrated here in a green-tech scenario, and thus may play a role in contributing toward the energy efficiency gap. Unfamiliarly with innovative technologies (such as heat-pumps) may lead consumers to feel they cannot simply focus on determining the objectively ‘best’ option in terms of alignable features, such as operational costs and expected energy savings. In real-world settings, we might otherwise expect a focus here to increase uptake of new, more energy-efficient systems and technologies. Yet, research has shown that consumers continually underinvest in new technologies, preferring familiar, but seemingly inferior alternatives on these objective performance measures (see Hafner, Elmes, & Read, 2019; Jager, 2006; Steg & Vlek, 2009). The current research provides evidence for a novel explanation for this effect; when faced with a choice of dissimilar goods, consumers may be more likely to consider both alignable and nonalignable features, stretching the limits of their cognitive capacity increasing their tendency to use simplification techniques, such as selecting more familiar products (see, Gilovich et al., 2002; Hafner, Elmes, & Read, 2019; Schwartz, 2000, 2004). The current research therefore provides evidence that alignability effects influence product choices, and may contribute to the continued prevalence of the energy efficiency gap for large-scale product purchase decisions. However, it is important to note that green decisions are not uniquely or disproportionately affected by alignability; rather, these results may apply to any choice context where the chooser is faced with unfamiliar, innovative alternatives that are outside of the norm.

Another key aim of the current research was to follow this line of reasoning through to determine the most effective way to structure the choice environment so as to promote ‘green choice’ of new technologies. Our results suggest that the key is to simplify the choice environment. For instance, if new heating system technologies are marketed as simply being a like-for-like replacement for a standard boiler, without presenting the chooser with too much information on unique functions or features, then this may increase likelihood of selection, if superior alignable attributes are simultaneously promoted. Indeed, supporting this suggestion, Jager (2006) suggests the emphasis on nonalignable features such as perceived complexity of installation and usage represent significant psychological barriers preventing uptake of photovoltaic (PV) panels in homes. What is more, Jager (2006) discusses how simplifying the presentation of new alternatives may facilitate wider integration and uptake. Crucially, this includes a focus on providing consumers with information on ease of use and emphasizing that minimal behavioral adjustments will be required in terms of daily operation on the part of the consumer. Our findings suggest that this may prove to be an effective longer term strategy for enabling positive behavior change and increasing uptake of innovative technologies. This is because by presenting options as a like-for-like replacement involving minimal effort or behavioral disruption on the part of the consumer, we may be able to reduce the emphasis that may otherwise be placed upon these nonalignable features. Thus, subjects may then be better able to focus on optimizing outcome in terms of alignable features such as operational costs and expected energy savings. Determining how this may be incorporated into marketing schemes in practice remains an interesting avenue for future research. In addition, it is important to note that these suggestions are based upon the findings of a hypothetical choice experiment, and subsequently more research will be needed to establish how much the results generalize to real-life settings.

However, it is also important to note that another potential explanation for the apparent split-shift in attentional focus when faced with a choice of dissimilar products stems from the fact that subjects may simply be more driven to make category-based judgments when options differ. Thus, a person who prefers green to nongreen products may simply have opted to choose the green alternative as a result of this category-based preference. A substantial body of research is consistent with the view that people can and do use category-based induction when category membership is known (see for instance, Murphy & Ross, 1994; Osherson, Smith, Wilkie, Lopez, & Shafir, 1990). However, we tentatively suggest that our results support the account that option preference remained the product of a feature-driven, as opposed to category-based inductive process. This is because we did not find any substantial overall difference in preference for the green versus nongreen product when options differed. If subjects were choosing simply on the basis of preexisting category-based judgments then, given general marketplace preference for nongreen products and previous research which has shown the majority of subjects will choose a nongreen product when feature alignability or choice frame information is *not* directly manipulated (see, for instance, Hafner, Elmes, Read, & White, 2019), we might have expected to see a greater proportion of subjects simply opting for the nongreen alternative. However, our results do not show this. The fact that we found no clear preference among the alternatives according to whether the products were green or not appears to support our account that subjects may in fact have been driven by feature-based reasoning, and thus may have experienced increased motivation to process all of the presented information at the moment of choice when options were dissimilar (vs. similar). This suggestion is further supported by evidence that subjects often prefer feature-based (vs. category-based) reasoning, even when categories with relatively high internal coherence are used (see, for instance, Griffiths, Hayes, & Newell, 2012).

We note, however, that because we included no measure of inductive reasoning strategy in our research, this suggestion remains speculative. Consequently, to gain a deeper and more definitive understanding of these effects, it would be useful for future research to conduct a replication experiment in which participants are asked to choose between green and conventional heating systems on the basis of category labels alone, that is, without features listed. If participants are then found to favor (or disfavor) the green option by a margin significantly different from that seen in the current data, this will provide direct evidence that the judgments were driven by feature information. If, however, evidence is then found for category-based judgments, the practical implications for encouraging uptake of green technologies may then center around exploration of the most effective means of recategorization of these alternatives (e.g., as instruments to achieve longer-term financial savings). This would likely be imperative given that at present, promotion of the environmental soundness or green-ness of a product alone as a means of categorization isn’t necessarily enough to drive product uptake (Hafner, Elmes, Read, & White, 2019; Jager, 2006; Steg & Vlek, 2009). This remains an interesting avenue for further research. However, given the lack of any clear preference for green versus conventional products when options differed in the current study, we tentatively suggest that our results appear to be more indicative of feature-based inductive process, but further research will be needed to substantiate this claim.

Overall then, we find evidence that attribute alignability influences preference formation and product choice and thus may contribute toward the continued prevalence of the energy efficiency gap in the context of large-scale purchase decisions. We have made suggestions for future research which may help to overcome these effects, by drawing attention instead back to superior alignable features associated with new technologies. Further research will also be needed to establish the parameters of alignability effects across varying choice contexts. However, the current research provides a useful step in developing understanding of how alignability effects may contribute to large-scale one-off purchase decisions. We suggest that these results should be carefully considered by those looking to develop strategies to reduce the energy efficiency gap in this context, ultimately taking us one step nearer to the goal of encouraging widespread uptake of nonstandard green systems and technologies.

## Figures and Tables

**Table 1 tbl1:** The Effects of Similarity on Mean Tendency to Infer Missing Information Is the Same, Collapsed Across Nonalignable Features (0 [Inferred Difference]–1 [Directly Copied])

Heating system task				Snack choice task			
Similar (boiler vs. boiler)		Dissimilar (boiler vs. heat pump)		Similar (popcorn vs. popcorn)		Dissimilar (popcorn vs. pretzels)	
*M*	*SD*	*M*	*SD*	*M*	*SD*	*M*	*SD*
.37	.24	.27	.18	.29	.21	.27	.16

## A Details of Attributes Used Interchangeably as Alignable or Nonalignable According to Accompanying Scales Provided, for the Heating Systems Task

**Table:**

Attribute	Scale	Description
Efficiency rating (% SAP 2009)	75; 85; 95	Measured by an independent laboratory, this is the measured energy efficiency of the appliance, including heat and hot water; it is calculated to an industry standard called SAP2009. This SAP rating is expressed on a scale of 1 to 100; the higher the number, the lower the running costs
Warranty (years)	2; 8; 15	
NOx emissions class	3; 4; 5	The official classification for the emission of the polluting gas NOx, class 5 is the best class with the lowest NOx emissions
Volume (liters)	70 (small); 200 (mid); 300 (large)	Volume including any space required for ventilation or cables
Product lifespan	5 years; 15 years; lifetime	
Brand reliability rating	★;★★★;★★★★★	Based on the results of an annual survey by Which? members
Hot water flow rate (liters/min)	9; 14; 18	How much hot water per minute the system is able to produce. The higher the number the more instant hot water you have available
Uptake in U.K.	Minimal (<2% of U.K. sales); moderate (approx. 5% of U.K. sales); extensive (approx. 10% of U.K. sales)	
Electricity usage on standby (W)	Minimal (2); Mid (6); High (10)	The amount of electricity used when the boiler is not firing
Ease of installation	Easy (takes around 1 hour); Moderate (takes up to 3 hours); Difficult (up to a whole working day)	
Temperature range	Limited (15°C–25°C); Average (12°C–28°C); Extensive (10°C–32°C)	
Availability of parts	Limited (specialist outlets only); Moderate (some major retailers); Widespread (most major retail outlets)	
Commonalities		
Concealed user controls	Yes; No	Are the user controls hidden behind a panel?
Timer?	Yes; No	Timer provided in user controls?
System pressure gauge?	Yes; No	Indicates the water pressure in the central heating system
Hot water temperature control?	Yes; No	

## B Details of Attributes Used Interchangeably as Alignable or Nonalignable According to Scales Provided, for the Snack Choice Task

**Table:**

Attribute	Scale
Piece size	Small; Medium; Large
Level of salt	Low; Moderate; High
Calorie content (per 100g)	Low (150); Mid (300); High (500)
Iron content (% RDA per 100g)	Low (16%); Mid 28%); High (44%)
Saturated fat (per 100g)	Low (.5g); Mid (4g); High (7g)
Satiety (fullness level)	Minimal; Moderate; High
Crunchiness	Not crunchy; Moderate; Very crunchy
Flavor strength	Weak; Moderate; Strong
Level of artificial additives	Low; Moderate; High
Carbohydrate content (per 100g)	Low (20g); Mid (50g); High (80g)
Popularity in the U.K.	Low (Less than .5% of U.K. sales); Moderate (Approx. 4% of U.K. sales); High (Approx. 9% of U.K. sales)
Quality of ingredients	Low (low quality/budget); Moderate (mix of high and low quality); High (organic/ethically sourced)
Commonalities	
Low cost per serving	Yes; No
Made from whole grain?	Yes; No
High in fiber?	Yes; No
Produced in the U.K.?	Yes; No
